# Immunological sub-phenotypes and response to convalescent plasma in COVID-19 induced ARDS: a secondary analysis of the CONFIDENT trial

**DOI:** 10.1186/s13613-024-01392-1

**Published:** 2024-10-21

**Authors:** Benoît Misset, Anh Nguyet Diep, Axelle Bertrand, Michael Piagnerelli, Eric Hoste, Isabelle Michaux, Elisabeth De Waele, Alexander Dumoulin, Philippe G. Jorens, Emmanuel van der Hauwaert, Frédéric Vallot, Walter Swinnen, Nicolas De Schryver, Nathalie de Mey, Nathalie Layios, Jean-Baptiste Mesland, Sébastien Robinet, Etienne Cavalier, Anne-Françoise Donneau, Michel Moutschen, Pierre-François Laterre

**Affiliations:** 1 Department of Intensive Care Medicine, Liège University, CHU de Liège, Liege, Belgium; 2 Biostatistic Unit, Public Health Department, Liège University, Liege, Belgium; 3 Department of Intensive Care Medicine, Marie-Curie University Hospital, Université Libre de Bruxelles, Charleroi, Belgium; 4 Department of Intensive Care Medicine, University Hospital, Ghent, Belgium; 5 Department of Intensive Care, Université Catholique de Louvain, CHU UCL Namur, Yvoir, Belgium; 6 Department of Clinical Nutrition, Vrije Universiteit Brussel Brussels University Hospital, Jette, Belgium; 7 Department of Intensive Care Medicine, Delta General Hospital, Roeselare, Belgium; 8 Department of Intensive Care Medicine, Antwerp University Hospital, University of Antwerp, LEMP, Edegem, Belgium; 9 Department of Intensive Care Medicine, Imelda General Hospital, Bonheiden, Belgium; 10 Department of Intensive Care Medicine, Wallonie Picarde General Hospital, Tournai, Belgium; 11 Department of Intensive Care Medicine, Sint Blasius General Hospital, Dendermonde, Belgium; 12 Department of Intensive Care Medicine, Saint-Pierre Medical Clinic, Ottignies, Belgium; 13 Department of Intensive Care Medicine, OLV General Hospital, Aalst, Belgium; 14 Department of Intensive Care Medicine, Saint-Luc University Hospital, Brussels, Belgium; 15 Department of Clinical Chemistry, University of Liege, CIRM, CHU de Liège, Liege, Belgium; 16 Department of Infectious Diseases, CHU de Liège, Liege, Belgium; 17 Department of Intensive Care Medicine, Mons-Hainaut Regional Hospital, Mons, Belgium; 18 Department of Intensive Care Medicine, University Hospital of Liège, Domaine Universitaire du Sart-Tilman, 4000 Liège, Belgium

**Keywords:** COVID-19, Convalescent plasma, ARDS, Immune response, Phenotypes

## Abstract

**Background:**

Convalescent plasma (CP) reduced the mortality in COVID-19 induced ARDS (C-ARDS) patients treated in the CONFIDENT trial. As patients are immunologically heterogeneous, we hypothesized that clusters may differ in their treatment responses to CP.

**Methods:**

We measured 20 cytokines, chemokines and cell adhesion markers using a multiplex technique at the time of inclusion in the CONFIDENT trial in patients of centers having accepted to participate in this secondary study. We performed descriptive statistics, unsupervised hierarchical cluster analysis, and examined the association between the clusters and CP effect on day-28 mortality.

**Results:**

Of the 475 patients included in CONFIDENT, 391 (82%) were sampled, and 196/391 (50.1%) had been assigned to CP. We identified four sub-phenotypes representing 89 (22.8%), 178 (45.5%), 38 (9.7%), and 86 (22.0%) patients. The most contributing biomarkers in the principal component analysis were IL-1β, IL-12p70, IL-6, IFN-α, IL-17A, IFN-γ, IL-13, TFN-α, total IgG, and CXCL10. Sub-phenotype-1 displayed a lower immune response, sub-phenotype-2 a higher adaptive response, sub-phenotype-3 the highest innate antiviral, pro and anti-inflammatory response, and adhesion molecule activation, and sub-phenotype-4 a higher pro and anti-inflammatory response, migration protein and adhesion molecule activation. Sub-phenotype-2 and sub-phenotype-4 had higher severity at the time of inclusion. The effect of CP treatment on mortality appeared higher than standard care in each sub-phenotype, without heterogeneity between sub-phenotypes (*p* = 0.97).

**Conclusion:**

In patients with C-ARDS, we identified 4 sub-phenotypes based on their immune response. These sub-phenotypes were associated with different clinical profiles. The response to CP was similar across the 4 sub-phenotypes.

*Trial registration*: Ethics Committee of the University Hospital of Liège CE 2020/239. Clinicaltrials.gov NCT04558476. Registered 2020-09-11, https://www.clinicaltrials.gov/study/NCT04558476.

**Supplementary Information:**

The online version contains supplementary material available at 10.1186/s13613-024-01392-1.

## Introduction

Acute respiratory distress syndrome (ARDS) was a prominent feature during the first three years of the COVID-19 pandemic, leading to hospital saturation all over the world [1]. Among therapies targeting the response against SARS-CoV-2 in these patients, low-dose steroids for 10 days was the first accepted therapy [2]. Passive immunization with plasma collected in COVID-19 convalescents was inconclusive at various stages of the disease [3, 4]. In patients admitted to the Intensive Care Unit (ICU) for SARS-CoV-2 induced pneumonia, the REMAP-CAP trialists observed a lower likelihood of providing improvement in organ support-free days [5]. In a secondary analysis, unsupervised analysis based on cytokines, chemokines and endothelial biomarkers, allowed to individualize sub-phenotypes with different response to convalescent plasma (CP) [6]. As severe COVID-19 has been considered as a particular form of sepsis [7] and/or ARDS [8], future trials should implement some form of predictive enrichment to increase the likelihood for beneficial effects of an intervention to emerge [9].

In the CONFIDENT trial of CP, we observed that patients with COVID-19 induced ARDS in the first days of invasive mechanical ventilation (IMV) experienced a significant reduction of mortality at 28 days [10]. By comparison to prior trials, we attributed this positive result to a greater homogeneity of the study population and to the high neutralizing activity of the CP we administered.

In the present study, as the individual immune response is likely heterogeneous, we hypothesized that the response to CP could differ in sub-groups determined by their immune profile. We tested this hypothesis as a secondary analysis in the patients of the CONFIDENT trial whose blood samples had been centralized for this purpose. The biomarkers we assessed were based on a multiplex test targeting 20 proteins involved in the inflammatory response. These proteins had all been described to be part of the immune response commonly observed in severe COVID-19 [11–18].

## Methods

### Study design

The present study is a secondary analysis of CONFIDENT and is labelled CONFIDENT-II. CONFIDENT was a publicly funded Belgian multicenter (17 centers) randomized open-label trial. The trial was designed to determine the effect on mortality at day-28 of CP with a neutralizing titer against SARS-CoV-2 at least 1/160 vs. standard care (SC) in patients with C-ARDS requiring invasive mechanical ventilation (IMV) for less than 5 days during the pandemic. The randomization process included a stratification based on the prior duration of IMV (≤ or > 48 h). The reduction of mortality was mainly observed in the patients who underwent randomization 48 h or less after IMV initiation.

### Study population

The CONFIDENT trial involved adult patients with a Clinical Frailty Scale < 6 [19], admitted to a participating ICU with a diagnosis of C-ARDS and submitted to IMV for a maximum of 5 days (WHO 10-point progression scale 7 to 9 [20]). ARDS was classified according to the Berlin definition [21]. C-ARDS was defined by an extended pneumonia on a CT scan or a chest X-ray within 10 days, and a positive result of SARS-CoV-2 nasopharyngeal PCR (NP-PCR) test within 15 days prior to inclusion. Exclusion criteria were pregnancy, prior episode of transfusion-related side effect, medical decision to limit therapy, and participation in another COVID-19 trial. The trial involved 475 patients between September 10, 2020, and March 9, 2022, and showed a 9.6% crude reduction in mortality at day 28 (*p* = 0.03).

The CONFIDENT-II population involved the patients who were included in those 12 centers having accepted to centralize blood samples for secondary analyses. Out of the trial population, plasma total IgG against SARS-CoV-2 and biomarkers were collected after patients’ informed consent and before randomization to CP or standard care (SC). The trial was registered at clinicaltrials.gov as NCT04558476 and approved by the institutional review boards of all centers.

### Biomarkers

The measurements were performed at the Laboratory Medicine Department of the CHU de Liège, Belgium. Total IgG against SARS-CoV-2 were assessed with the LIAISON® SARS-CoV-2 TrimericS IgG chemiluminescent kit (Diasorin, Saluggia, Italy). Results are expressed as Binding Antibody Units per mL (BAU/mL). A value > 33.7 BAU/mL is considered positive (manufacturer). Values of 535, 606, 860 and 1335 BAU/mL are considered predictive of 20, 40, 160 and 320 neutralizing antibody titers with 50% plaque reduction (PRNT50), respectively [22].

The balance between pro- and anti-inflammatory mediators was assessed using Luminex xMAP technology with the Human Inflammation ProcartaPlex™ Panel (20-Plex, Thermo Fisher Scientific, MA). The biomarkers assessed were thirteen cytokines (GM-CSF, IFN-α, IFN-γ, IL-1α, IL-1β, IL-4, IL-6, IL-8, IL-10, IL-12p70, IL-13, IL-17A (CTLA-8), and TNF-α), four chemokines (IP-10 (CXCL10), MCP-1 (CCL2), MIP-1α (CCL3), and MIP-1β (CCL4)), and three cell adhesion molecules (ICAM-1, CD62E (E-selectin), and CD62P (P-Selectin)). All the samples were measured in duplicate and the coefficients of variation were all <15%. The results were provided as quantitative values in pg/mL[23]. For biomarkers whose value was below the lower limit of quantification (LLoQ), we attributed a value of LLoQ. For biomarkers whose value was over the upper limit of quantification (ULoQ), we attributed the ULoQ value. Blood CRP and platelets were included among the biomarkers as indicators of both inflammation and coagulation activation [24].

### Statistics

Variables are provided as mean (SD) or median (IQR) or counts (percentages). The homogeneity between the CONFIDENT and CONFIDENT-II populations was estimated on baseline characteristics and the primary endpoint (day-28 mortality). Comparisons were made with the Chi-square Fisher’s exact, and *t*-test or and Mann–Whitney tests if assumptions regarding the two tests were not satisfied. The effect on the principal endpoint was measured with the Odds ratio (OR).

### Cluster analysis

Agglomerative hierarchical cluster analysis was applied to examine the number of clusters of participants in the included cohort explained by the biomarkers. We chose this unsupervised technique because the number and the definition of the clusters based on immune response are not known a priori in the setting of sepsis, ARDS and/or CIVD-19. Wald.D2 linkage function was employed to investigate the variance of the clusters on the log10-transformed biomarker data. The number of clusters were determined based on the dendogram and the elbow method or the total within sum of squared distances (SSE) [25]. Accordingly, the SSE between each observation and the centroid respective to the cluster to which the observation was assigned as a result of clustering. K (numbers of clusters) was set from 1 to 10 and the SSE of each observation to the corresponding closest centroid was calculated for each value of k. The results were presented by means of an elbow plot. The point where the SSE decreased sharply or leveled off was determined as the optimal value for k. Principal component analysis (PCA) was performed to determine the most contributing biomarkers. Based on descriptive statistics, we normalized the protein biomarkers to the median of the sub-phenotype with the lowest median values for most biomarkers, namely sub-phenotype-1, and presented with boxplots of log2 fold-change.

### Clusters and treatment effect

We used Kruskal Wallis, chi-square and/or Fisher’s exact tests to examine the differences among the identified clusters with regards to clinical characteristics. We examined differences in the CP treatment effect as to D-28 mortality within each cluster and as a whole cohort. A test of heterogeneity of the ORs was performed and presented with the Q-statistic. The results were visualized by means of a forest plot, displaying the ORs and the respective 95% confidence interval (CI).

The analyses were performed with R statistical software, version 4.2.2 (R Core Team, 2021).

## Results

### Study cohort

**Fig. 1:**
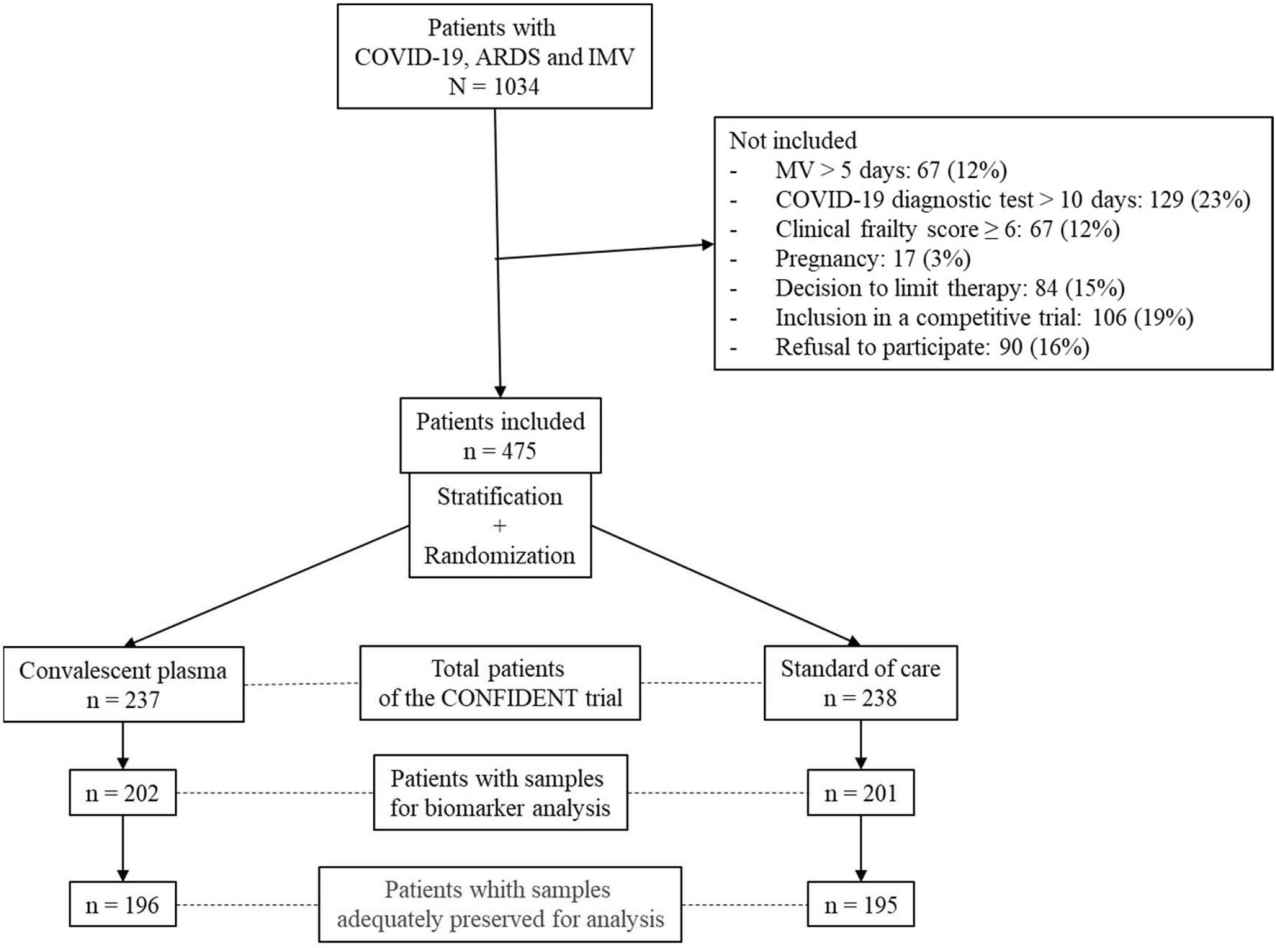
Flowchart of the CONFIDENT-II cohort

Among the 475 participants in the CONFIDENT trial [10], 237 patients were randomly assigned to CP and 238 to SC. Twelve of 17 (70%) centers collected samples in 403/475 (85%) patients for secondary analyses, 202 assigned to CP and 201 to SC. The biological samples could not be used in 12 patients due to a lack of preservation during storage, resulting in a total of 391/475 (82%) patients who could be analyzed in the CONFIDENT-II cohort (Fig. 1). Of these, 196 (50.1%) had been assigned to CP and 195 (49.9%) to SC with a median age was 64 [IQR: 56–72] years. 66.8% (n = 261) were males. Most participants had blood group A (n = 180, 46.0%) and O (n = 158, 40.4%), their median APACHE II score was 13 [IQR: 9–17], with a P/F ratio 116 [89–174]. In this secondary analysis, 375 (95.9%) patients were receiving steroids; and 299 (76.5%) were included within 48 h of IMV. 149 (38.1%) patients died before day-28. The characteristics of the CONFIDENT-II patients were similar to those in the CONFIDENT cohort except for a lower PaO2/FiO2 ratio and a shorter delay from ICU admission to inclusion in the study cohort. The characteristics of the CP and SC groups within the CONFIDENT-II population are similar (Table 1).

**Table 1:** Characteristics of the study population

	CONFIDENT-II population			Population not included			CONFIDENT population	*p*-value
	Convalescent (n = 196)	Standard (n = 195)	Overall (n = 391)	Convalescent (n = 41)	Standard (n = 43)	Overall (n = 84)	Total (n = 475)	
Age, years	64 [56–72]	65 [56–71]	64 [56–72]	64 [52–70]	63 [56–72]	63.5 [55–70]	64 [56–72]	0.38
Male sex, n	130 (66.3)	131 (67.2)	261 (66.8)	28 (68.3)	34 (79.1)	62 (73.8)	323 (68.0)	0.26
IMV < 48 h at inclusion, n	150 (76.5)	149 (76.4)	299 (76.5)	21 (51.2)	22 (51.2)	43 (51.2)	342 (72.0)	<0.001
BMI, kg/m^2^	30.5 [26.4–34.8]	29.9 [26.5–34.2]	30.4 [26.4–34.5]	30.4 [27.2- 34.5]	29.4 [26.4–37.1]	29.7 [27.0–35.9]	30.2 [26.5–34.6]	0.73
Blood group, n								0.95
A	87 (44.6)	93 (47.4)	180 (46.0)	23 (56.1)	16 (37.2)	39 (46.4)	219 (46.1)	
AB	8 (4.1)	6 (3.1)	14 (3.6)	1 (2.4)	1 (2.3)	2 (2.4)	16 (3.4)	
B	19 (9.7)	20 (10.2)	39 (10.0)	4 (9.8)	5 (11.6)	9 (10.7)	48 (10.1)	
O	81 (41.5)	77 (39.3)	158 (40.4)	13 (31.7)	21 (48.8)	34 (40.5)	192 (40.4)	
NP PCR test for SARS-Cov-2, Ct	21 [18–26]	20 [17–25]	21 [18–26]	23 [19–27]	23 [16–27]	23 [19–27]	21 [17–26]	0.41
Time from ICU admission, days	2.6 [1.6–4.6]	3.4 [1.7–5.1]	2.7 [1.6–4.6]	4.4 [2.7–5.8]	4.5 [3.5–5.6]	4.4 [3.4–5.7]	3.4 [1.7–4.7]	<0.001
Severity at ICU admission								
APACHE II score, points	13 [9–18]	13 [9.25–17]	13 [9–17]	14 [9–17]	12 [8–15]	12 [8.25–16]	13 [9–17]	0.38
SOFA, points	6 [4–8]	6 [4–8]	6 [4–8]	6 [5–8]	6 [4–8]	6 [4–8]	6 [4–8]	0.96
PEEP level, mmHg	10 [10–12]	10 [10–12]	10 [10–12]	10 [10–13]	12 [8–13]	10 [9.9–13]	10 [10–12]	0.59
PaO2/FiO2, mmHg	116 [89–154]	124 [91–155]	116 [89–154]	129 [99–174]	145 [106–172]	137 [100–174]	123 [91–160]	0.03
CRP, mg/L	125 [67–189]	115 [65–198]	123 [65–191]	130 [65–214]	101 [43–150]	123 [55–189]	123 [91–160]	0.18
WHO progression scale	8 [8–8]	8 [8–8]	8 [8–8]	8 [7–8]	8 [7–8]	8 [7–8]	8 [8–8]	0.02
Comorbidities, n								
Hypertension	121 (61.7)	104 (53.3)	225 (57.5)	24 (58.5)	25 (58.1)	49 (58.3)	274 (57.7)	0.90
Congestive heart failure	15 (7.7)	9 (4.6)	24 (6.1)	4 (9.8)	2 (4.7)	6 (7.1)	30 (6.3)	0.88
COPD	23 (11.7)	22 (11.3)	45 (11.5)	4 (9.8)	2 (4.7)	6 (7.1)	51 (10.7)	0.34
Asthma	20 (10.2)	17 (8.7)	37 (9.5)	2 (4.9)	0 (0)	2 (2.4)	39 (8.2)	0.06
Diabetes	68 (34.7)	73 (37.4)	141 (36.1)	13 (31.7)	19 (44.2)	32 (38.1)	173 (36.4)	0.77
Chronic renal failure	26 (13.3)	24 (12.3)	50 (12.8)	6 (14.6)	6 (14.0)	12 (14.3)	62 (13.1)	0.82
Haematological_cancer	4 (2.0)	10 (5.1)	14 (3.6)	2 (4.9)	1 (2.3)	3 (3.6)	17 (3.6)	1.00
Solid tumor	6 (3.1)	12 (6.2)	18 (4.6)	0 (0)	1 (2.3)	1 (1.2)	18 (3.8%)	0.09
Therapy against SARS-Cov-2, n								
Hydroxychloroquine	1 (0.5)	0 (0)	1 (0.3)	0 (0)	0 (0)	0 (0)	1 (0.2)	1.00
Azythromycin	6 (3.1)	3 (1.5)	9 (2.3)	4 (9.8)	1 (2.3)	5 (6.0)	14 (2.9)	0.15
Remdesivir	8 (4.1)	12 (6.2)	20 (5.1)	5 (12.2)	2 (4.7)	7 (8.3)	27 (5.7)	0.37
Anti-IL-6 or IL-6R	10 (5.1)	5 (2.6)	15 (3.8)	2 (4.9)	2 (4.7)	4 (4.8)	19 (4.0)	0.94
Any steroid	192 (98.0)	191 (97.9)	383 (98.0)	41 (100.0)	42 (97.7)	83 (98.8)	466 (98.1)	1.00
Death at day-28, n	70 (35.7)	79 (40.5)	149 (38.1)	14 (34.1)	28 (65.1)	42 (50.0)	191 (40.2)	0.06

*IMV* invasive mechanical ventilation, *BMI* body mass index, *NP-PCR* naso-pharyngeal polymerase chain reaction, *SARS-CoV-2* severe acute respiratory syndrome coronavirus 2, *ICU* intensive care unit, *APACHE II* Acute Physiology And Chronic Health Evaluation II, *SOFA* sequential organ failure assessment, *PEEP* positive end-expiratory pressure, *PaO2* arterial partial pressure of oxygen, *FiO2* fraction of inspired oxygen, *CRP* C-reactive protein, *WHO* world health organization, *COPD* chronic obstructive pulmonary disease, *IL-6* interleukin-6, *IL-6R* IL-6 receptor

The frequency of missing samples was less than 1% for all the data presented, except for BMI (6.9%) and for the quantitative value of the SARS-CoV-2 nasopharyngeal PCR (NP-PCR) patients (32.2%) because the routine laboratory of several centers responded a qualitative (yes/no) or semi-quantitative result. We considered that these missing values were completely at random

The frequency of missing samples was less than 1% for all the data presented, except for BMI (6.9%) and for the quantitative value of the SARS-CoV-2 NP-PCR patients (32.2%) because the routine laboratory of several centers responded a qualitative (yes/no) or semi-quantitative result. Therefore, we considered that these missing values were completely at random.

### Clusters

**Fig. 2:**
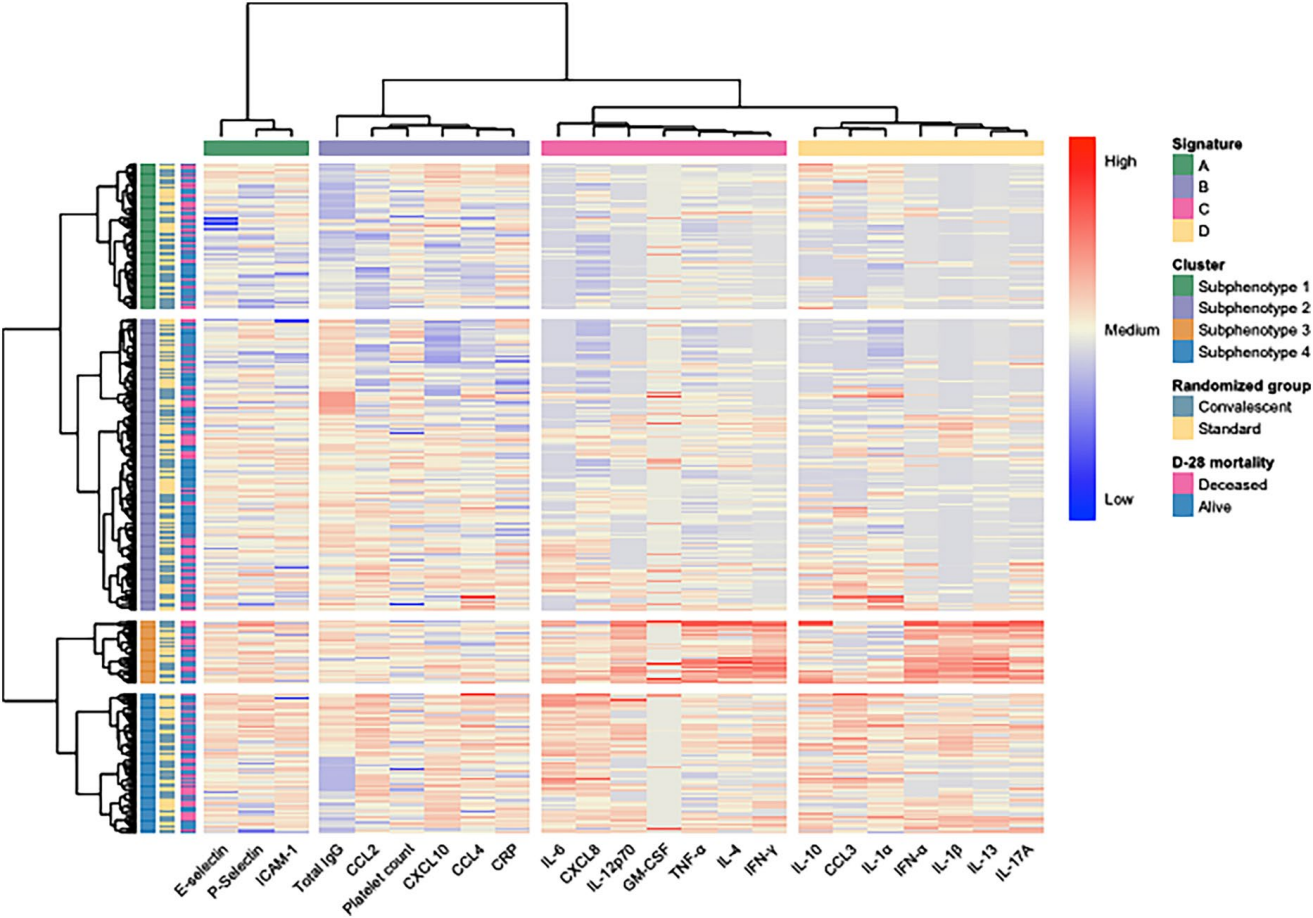
Heatmap of the sub-phenotypes and biomarker signatures in C-ARDS patients. Each patient (n = 391) is represented by a row. The rows are grouped into 4 patients’ sub-phenotypes. The first column indicates the subphenotype of the patients. The second column indicates the group of randomization of the patients. The third column indicates the day-28 mortality. The following 22 columns are grouped into 4 biomarker signatures (*A*–*D*). The biomarker signatures, sub-phenotype numbers, treatment groups, and mortality at D-28 are noted using a color code provided on the right legend of the figure. Each biomarker (n = 23) level transformed into a log10 scale is indicated in the following columns and represented by a color gradient going from *blue* (low level) to *red* (high level)

**Fig. 3:**
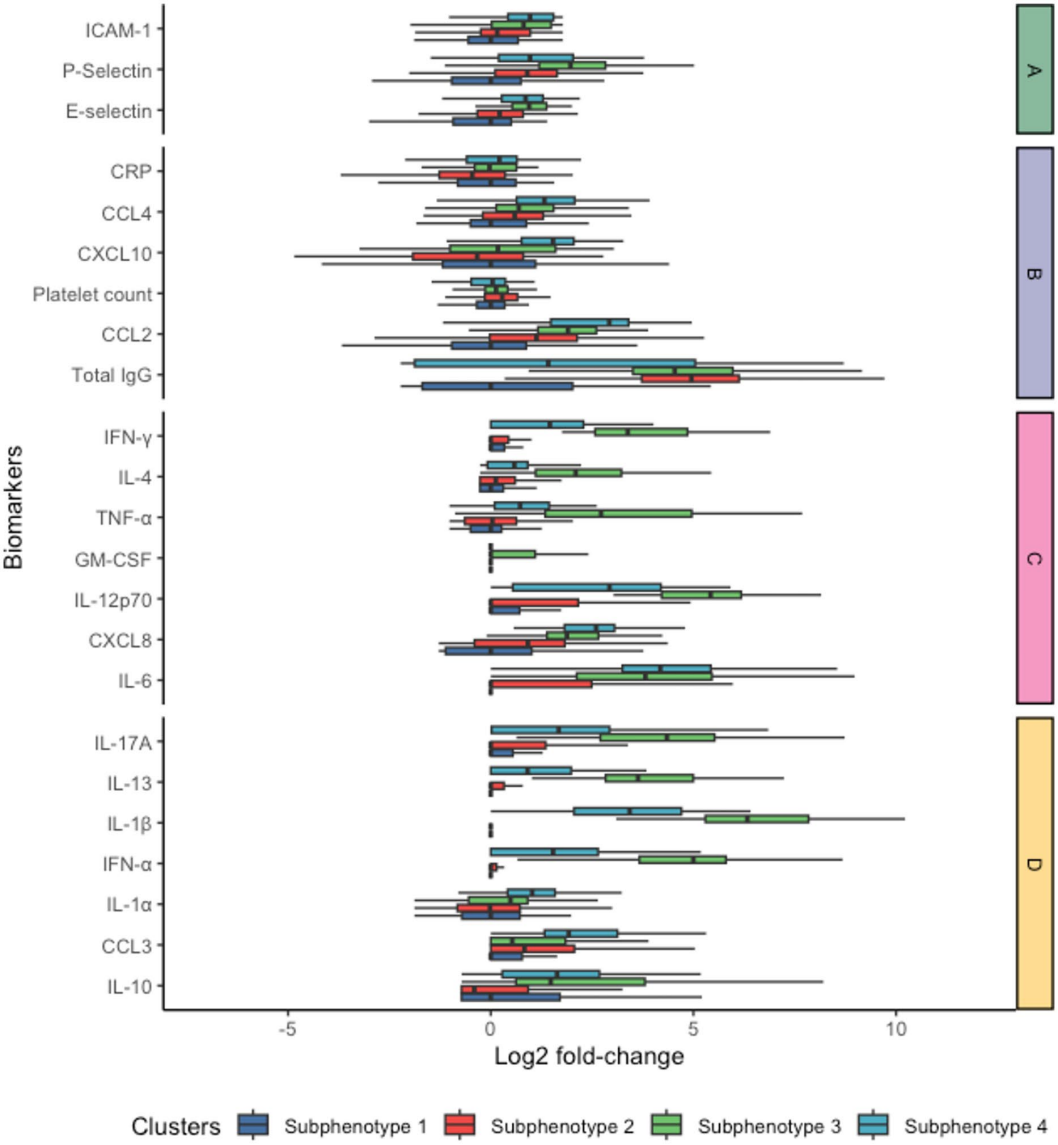
Box-and-whisker plots of biomarkers according to sub-phenotypes. Biomarker values are log2 transformed and normalized to the median of sub-phenotype 1 (if the difference of the two log2 of a biomarker of 2 subphenotypes, such as sub- phenotype 2 and sub- phenotype 1, is 1, this means that sub-phenotype 2 has a level twice as high as sub-phenotype 1). They are grouped by biomarker signature (*A*–*D*). Boxes are colored by sub-phenotype (bottom legend of the figure). The bottom border of each box represents the 25th percentile; the line bisecting the box represents the median; the upper border of the box is the 75th percentile. The whiskers represent extremes, 1.5 times the 75th (highest) and 25th (lowest) values

The dendogram obtained from Hierarchical clustering suggested either a two or four-cluster approach. Examination of the elbow plot revealed that the value of k where the SSE dropped was 4 (e-Fig. 1). On this basis, we determined 4 biomarker signatures and 4 sub-phenotypes of patients that could be distinguished by their biomarker and clinical profiles grouped by 4 biomarker signatures (Figs. 2 and 3). The comparative blood levels of each biomarker in the sub-phenotypes are provided in e-Table 1.

Biomarker signatures were labelled A, B, C and D. Signature A mainly gathered cell adhesion markers (E-selectin, P-selectin, ICAM-1); signature B adaptive response (total IgG against SARS-CoV-2) and migration markers (CCL2, CXCL10, CCL4), signature C pro (IL-6, IL-12p70, GM-CSF, TNF-α, IFN-γ) and anti-inflammatory (IL-4) cytokines; and signature D innate cytokines (IFN-α), pro-antiviral response (IL-1α, IL-1β, IFN-α, IL17A), and anti-inflammatory cytokines (IL-10, IL-13).

Sub-phenotype-2 consisted of the most patients (n = 178, 45.5%), followed by sub-phenotype-1 (n = 89, 22.8%) and sub-phenotype-4 (n = 86, 22.0%), and sub-phenotype-3 (n = 38, 9.7%) (Table 2 and Fig. 2). The most contributing biomarkers were IL-1β, IL-12p70, IL-6, IFN-α, IL-17A, IFN-γ, IL-13, TFN-α, total IgG, and CXCL10 (e-Figs. 2, 3 and 4).

**Table 2:** Clinical characteristics of the sub-phenotypes

	Subphenotype 1	Subphenotype 2	Subphenotype 3	Subphenotype 4	Total	*p*-value
	(n = 89)	(n = 178)	(n = 38)	(n = 86)	(n = 391)	
Age, years	65 [56–72]	66 [56–73]	65 [59–72]	63 [57–70]	64 [56–72]	0.60
Male gender, n	64 (72.9)	114 (64.0)	27 (71.1)	56 (65.1)	261 (66.8)	0.56
BMI, kg/m^2^	30.8 [26.9–34.6]	30.7 [27.4–34.4]	29.2 [26.0–34.6]	28.2 [24.7–34.4]	30.4 [26.4–34.5]	0.08
ABO blood group, n						0.27
* A*	48 (53.9)	78 (43.8)	17 (44.7)	37 (43.0)	180 (46.0)	
* AB*	1 (1.1)	9 (5.1)	0 (0)	4 (4.7)	14 (3.6)	
* B*	13 (14.6)	15 (8.4)	3 (7.9)	8 (9.3)	39 (10.0)	
* O*	27 (30.3)	76 (42.7)	18 (47.4)	37 (43.0)	158 (40.4)	
NP PCR test for SARS-Cov-2, Ct	20 [18–24]	22 [19–28]	21 [18–25]	19 [16–22]	20 [18–38]	0.006
Time from ICU admission, days	2.5 [1.6- 3.4]	3.6 [2.4–5.4]	3.5 [1.6–5.5]	2.5 [1.5–4.5]	2.7 [1.6 – 4.6]	<0.001
Severity at inclusion						
IMV < 48 h, n	72 (80.9)	118 (66.3)	32 (84.2)	77 (89.5)	299 (76.5)	<0.001
APACHE II score, points	13 [9–18]	12 [9–16]	14 [11–18]	15 [12–19]	13 [9–17]	<0.001
SOFA total, points						
* Total*	6 [4–7]	6 [4–7]	7 [4–8]	7 [5–9]	6 [4–8]	<0.001
* Respiratory*	3 [3, 4]	3 [3, 4]	4 [4 - 4]	4 [3, 4]	4 [3, 4]	<0.001
* Coagulation*	0 [0–0]	0 [0–0]	0 [0–0]	0 [0–0]	0 [0–0]	0.17
* Liver*	0 [0–0]	0 [0–0]	0 [0–0]	0 [0–0]	0 [0–0]	0.28
* Cardiovascular*	1 [0–3]	1 [0–3]	3 [0–3]	3 [1–4]	3 [0–3]	0.001
* Central nervous system*	0 [0–0]	0 [0–0]	0 [0–0]	0 [0–0]	0 [0–0]	0.35
* Renal*	0 [0–0]	0 [0–0]	0 [0–0]	0 [0–1]	0 [0–0]	0.12
PaO2/FiO2, mmHg	138 [105–164]	122 [96–159]	96 [83–137]	98 [84–133]	116 [89–154]	<0.001
PEEP level, mmHg	10 [9–12]	10 [9–12]	10 [10–12]	10 [10–12]	10 [10–12]	0.54
WHO progression scale, points	8 [8–8]	8 [8–8]	8 [8–8]	8 [8–8]	8 [8–8]	0.47
Comorbidities, n						
Hypertension	51 (57.3)	102 (57.3)	26 (68.4)	46 (53.5)	225 (57.5)	0.49
Congestive heart failure	6 (6.7)	7 (3.9)	5 (13.2)	6 (7.0)	24 (6.1)	0.18
Diabetes	33 (37.1)	66 (37.1)	14 (36.8)	28 (32.6)	141 (36.1)	0.89
COPD	14 (15.7)	24 (13.5)	3 (7.9)	4 (4.7)	45 (11.5)	0.08
Asthma	8 (9.0)	18 (10.1)	3 (7.9)	8 (9.3)	37 (9.5)	0.97
Chronic renal failure	12 (13.5)	19 (10.7)	6 (15.8)	13 (15.1)	50 (12.8)	0.69
Hematologic cancer	4 (4.5)	2 (1.1)	0 (0.0)	8 (9.3)	14 (3.6)	0.005
Solid tumor	3 (3.4)	8 (4.5)	1 (2.6)	6 (7.0)	18 (4.6)	0.72
Therapy against SARS-Cov-2, n						
Hydroxychloroquine	0 (0.0)	0 (0.0)	0 (0.0)	1 (1.2)	1 (0.3)	0.32
Azythromycin	3 (3.4)	6 (3.4)	0 (0.0)	0 (0.0)	9 (2.3)	0.23
Remdesivir	8 (9.0)	7 (3.9)	2 (5.3)	3 (3.5)	20 (5.1)	0.30
Anti-IL-6 or anti-IL-6R	0 (0.0)	6 (3.4)	2 (5.3)	7 (8.1)	15 (3.8)	0.02
Dexamethasone	85 (95.5)	171 (96.1)	36 (94.7)	83 (96.5)	375 (95.9)	0.21
Treatment group and outcome, n						
Allocated to CP	49 (55.1)	85 (47.8)	20 (52.6)	42 (48.8)	196 (50.1)	0.70
Deceased at day-28	34 (38.2)	60 (33.7)	17 (44.7)	38 (44.2)	149 (38.1)	0.32

*IMV* invasive mechanical ventilation, *BMI* body mass index, *NP-PCR* naso-pharyngeal polymerase chain reaction, *SARS-CoV-2* severe acute respiratory syndrome coronavirus 2, *ICU* intensive care unit, *APACHE II* Acute Physiology And Chronic Health Evaluation II, *SOFA* sequential organ failure assessment, *PEEP* positive end-expiratory pressure, *PaO2* arterial partial pressure of oxygen, *FiO2* fraction of inspired oxygen, *WHO* world health organization, *COPD* chronic obstructive pulmonary disease, *IL-6* interleukin-6, *IL-6R* IL-6 receptor

The frequency of missing samples was less than 1% for all the data presented, except for BMI (6.9%) and for the quantitative value of the SARS-CoV-2 nasopharyngeal PCR (NP-PCR) patients (32.2%) because the routine laboratory of several centers responded a qualitative (yes/no) or semi-quantitative result. We considered that these missing values were completely at random

The *p*-value indicates the significance of the test comparing all the subphenotypes. Post-hoc test of significance between each subphenotypes is provided in e-Table 2

**Fig. 4:**
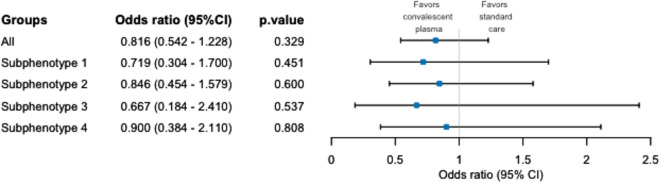
Forest plot describing the effect on mortality at day-28 of CP per sub-phenotype. *CI* confidence interval; the reference group to calculate Odds ratios is standard of care (SOC)

Sub-phenotype 1 was characterized by low IgG level and P-Selectin; and various levels of migration markers (CCL2, CXCL10, CCL4), as well as rather low reaction in biomarkers signature C. In general, the median values of most biomarkers were lower in sub-phenotype 1 than in other sub-phenotypes.

Sub-phenotype 2 demonstrated a high level of IgG, suggesting a higher adaptive response. Yet, just as sub-phenotype 1, patients in this group presented various levels of CRP, CCL3 and IL-1α.

Compared to the sub-phenotypes 1 and 2, sub-phenotype 3 was more homogeneous, demonstrating elevated levels of E-selectin, and P-selectin, Signature C pro (IL-12p70, TNF-α, IFN-γ, IL-4) and pro-antiviral response (IL-1β, IFN-α, IL17A) and anti-inflammatory cytokines (IL-13). Altogether, sub-phenotype 3 represented a higher innate antiviral, pro and anti-inflammatory response, and adhesion molecule activation.

Similarly, sub-phenotype 4 was distinguished from others by higher levels of cell adhesion markers (ICAM-1), migration markers (CCL2, CXCL10, CCL4), signature C pro (IL-6) and anti-inflammatory cytokines (IL-10), suggesting a higher pro and anti-inflammatory response, migration protein and adhesion molecule activation) (Figs. 2 and 3). These profiles are consistent with the immune profiles described in COVID-19 [12, 18].

By comparison to the other sub-phenotypes (Table 2 and Fig. 3), sub-phenotype 1 had a lower respiratory severity as expressed by a higher PaO2/FiO2 ratio; sub-phenotype 2 patients were included later in the disease course (they were more frequently under IMV for 2–5 days) and had higher circulating platelet counts. These 2 sub-phenotypes had a lower severity in terms of APACHE II and SOFA scores, and PaO2/FiO2 ratio than sub-phenotypes 3 and 4. Sub-phenotype 4 had higher circulating CRP levels. The post-hoc test of significance between each sub-phenotypes is provided in e-Table 2.

The patients in the 4 sub-phenotypes did not differ regarding age, gender, ABO blood group, BMI and prior length of hospital stay. Their severity at inclusion as assessed by APACHE II and SOFA scores was higher in the sub-phenotypes 3 and 4. Comorbidities were similar across the phenotypes except that prior hematological malignancy was more prominent in sub-phenotype 4. The use of concomitant medications against SARS-CoV-2 was similar across the sub-phenotypes, except that anti-IL-6 or IL-6R drugs were more frequently administered to the patients of sub-phenotype 4. The patients of sub-phenotype 4 were more frequently included in the first 48 h from the start of invasive ventilation, suggesting either a faster disease kinetics or a later presentation. The allocation of the trial intervention—CP or SC—was similar across the 4 sub-phenotypes (Table 2).

### Clusters and treatment effect

As in the entire population of the trial [10], the results favor CP over SC in all 4 sub-phenotypes. The OR in each sub-phenotype did not reach statistical significance. The heterogeneity of the response to CP between the four sub-phenotypes was insignificant (Q = 0.24, df = 3, *p* = 0.97) (Fig. 4 and e-Table 3).

## Discussion

Exploring the immunological response of patients presenting with C-ARDS and requiring IMV, we identified 4 biomarker signatures and 4 sub-phenotypes of patients with different immune profiles. These sub-phenotypes were associated with differences in terms of acute severity and kinetics of the disease, and their response to CP was consistent with its positive effect in the CONFIDENT trial [10] and did not differ between the phenotypes.

The differences in severity of the sub-phenotypes are consistent with an association between clinical severity and some modes of host defense [9]. The mortality was however not different, potentially because the number of patients included was too low for these scores to discriminate [26].

We have chosen biomarkers which make it possible to qualify the immune response during sepsis [27] as well as during COVID-19 [18]. We used a multiplex panel which is proposed to explore the “immune dysregulation” observed in sepsis [28] because it includes molecules describing the syndrome proposed under the term “cytokine storm” in COVID-19 [29]. These include mediators which amplify inflammation as well as the anti-inflammatory response. The regulation of this simultaneous response is supposed to make it possible to eliminate the pathogen while avoiding the dangers of an excessive inflammation. Additionally, a series of chemokines, have the particularity of facilitating the migration of effector cells in the body and certainly participate in the compartmentalization of the anti-infectious response [30]. Our panel of markers also includes markers of cell adhesion, an indicator of endothelial activation and coagulation [28]. The production of these biomarkers characterized the various responses observed in bacterial sepsis and in COVID-19 [18, 28]. Finally, our panel included alpha interferon, which is known to act in the innate anti-viral response [14].

The fact that we were able to identify clusters based on these biomarkers with unsupervised statistical analysis techniques was expected because this has already been observed in sepsis [31] and in COVID-19 [6]. This requires large patient cohorts and good clinical and pathophysiological homogeneity. These characteristics were observed during the first years of the COVID-19 pandemic. The different profiles that we observed are compatible with what has already been published during covid [6, 12, 13, 18].

When we initiated this secondary analysis, we assumed that we could individualize certain clusters with a better response to CP and confirm other results [6]. This was not the case, and we believe this may be due to several reasons. First, our cohort is more homogeneous than Fish’s population [6] in terms of the infection kinetics because our patients were all recruited within a narrow time range when ARDS appeared. This may have led to too small differences between the groups of patients identified by sub-phenotypes. Second, the panel of biomarkers that we used studies the innate immune and inflammatory response to viral infection, while CP is supposed to act via antibodies neutralizing SARS-CoV-2 [32] by reducing the quantity of viable virus in infected tissues, in this case the lung parenchyma. This action is located upstream of the inflammatory response. Therefore, it may have an impact whatever the inflammatory response secondary to the viral infection. This is consistent with the delay in effect on mortality of approximately 15 days that we observed with CP during ARDS in the CONFIDENT trial, the same delay as that between viral infection and appearance of ARDS [10]. Third, circulating factors may not reflect the tissue conditions. Sampling tissues was not done because clustering these patients was a secondary objective of the trial. We considered tissue sampling such as lung was excessively invasive for the patients. Fourth, while we used a multiplex kit that includes the most frequently biomarkers of sepsis, we may have missed certain important ones. Last, the lack of a statistical difference between subphenotypes for their response to convalescent plasma may be due to a type II error. Indeed, the calculation of the number of subjects needed to perform the confidant trial was determined on the primary endpoint and not on the secondary analyses.

## Conclusion

In a cohort of patients with C-ARDS included in a CP trial, we identified 4 sub-phenotypes based on their immune response. These sub-phenotypes were associated with different clinical profiles. The response to CP, as assessed by the day-28 mortality, was similar across the 4 sub-phenotypes.

## Supplementary Information

Additional file 1.

Additional file 2.

Additional file 3.

Additional file 4.

Additional file 5.

Additional file 6.

Additional file 7.

## Data Availability

The datasets used and/or analysed during the current study are available from the corresponding author on reasonable request.

## Abbreviations

TNF-α: Tumor necrosis factor alpha
IL-1α: Interleukin 1 alpha
IL-1β: Interleukin 1 alpha
IL-6: Interleukin 6
IL-12p70: Heterodimeric 70-kDa Interleukin 12
IL-17A: Interleukin 17A (also Interleukin 17 or CTLA8 (cytotoxic T-lymphocyte-associated antigen 8)
GM-CSF: Granulocyte-macrophage colony-stimulating factor
IFN-γ: Interferon gamma
IL-4: Interleukin 4
IL-10: Interleukin 10
IL-13: Interleukin 13
MCP-1: Monocyte chemoattractant protein 1 (C–C motif chemokine ligand 2 (CCL2))
MIP-1α: Macrophage inflammatory protein 1 alpha (C–C motif chemokine ligand 3 (CCL3))
MIP-1β: Macrophage inflammatory protein 1 beta (C–C motif chemokine ligand 4 (CCL4))
IL-8: Interleukin 8 (C-X-C motif chemokine ligand 8 (CXCL8))
IP-10: Interferon gamma-induced protein 10 (C-X-C motif chemokine ligand 10 (CXCL10))
ICAM-1: Intercellular Adhesion Molecule 1 (Cluster of Differentiation 54 (CD54))
CD62E: CD62 antigen-like family member E (E-selectin)
CD62P: CD62 antigen-like family member P (P-selectin)
IFN-α: Interferon alpha

## Declarations

### Clinical Trial Registration

Ethics Committee of the University Hospital of Liège CE 2020/239. Clinicaltrials.gov NCT04558476.
