# Supplementary figures and images for: Immunological sub-phenotypes and response to convalescent plasma in COVID-19 induced ARDS: a secondary analysis of the CONFIDENT trial
Source: Ann Intensive Care. 2024 Oct 21;14:160. doi: 10.1186/s13613-024-01392-1 (PMC11493925; doi:10.1186/s13613-024-01392-1)

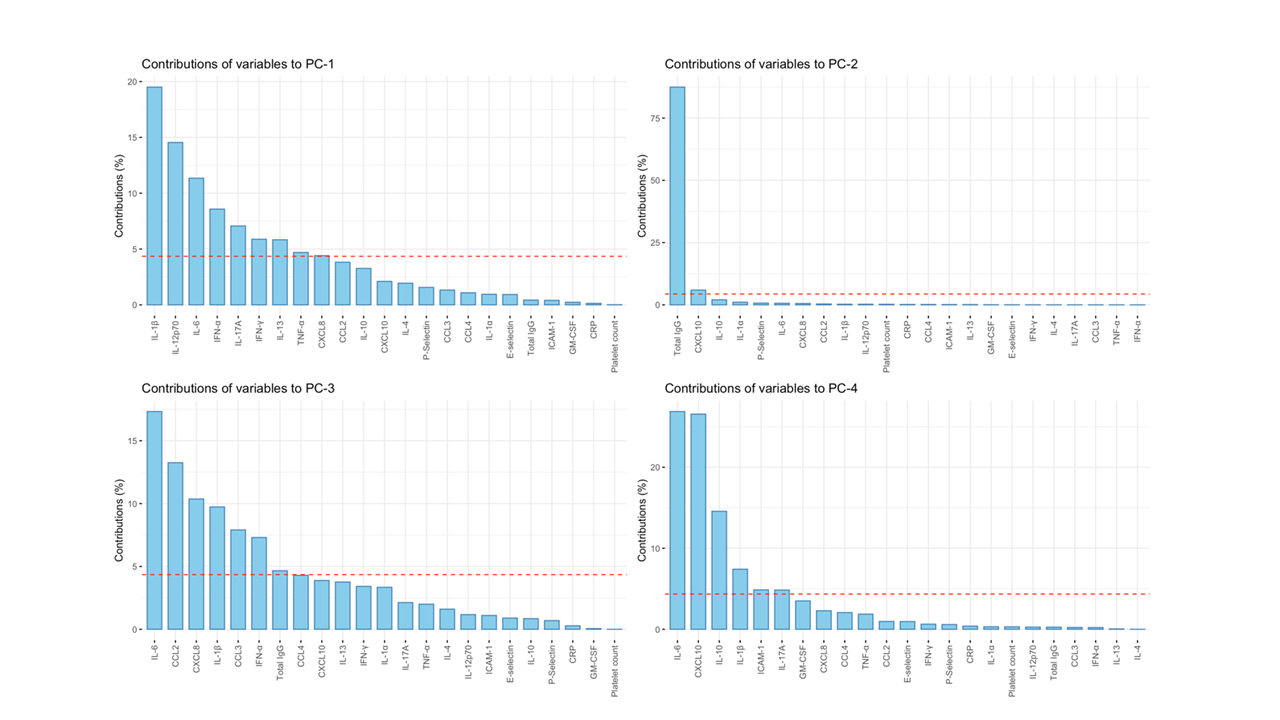

Supplement: Supplementary file 2 — Additional file 2. [file 13613_2024_1392_MOESM2_ESM.tif]

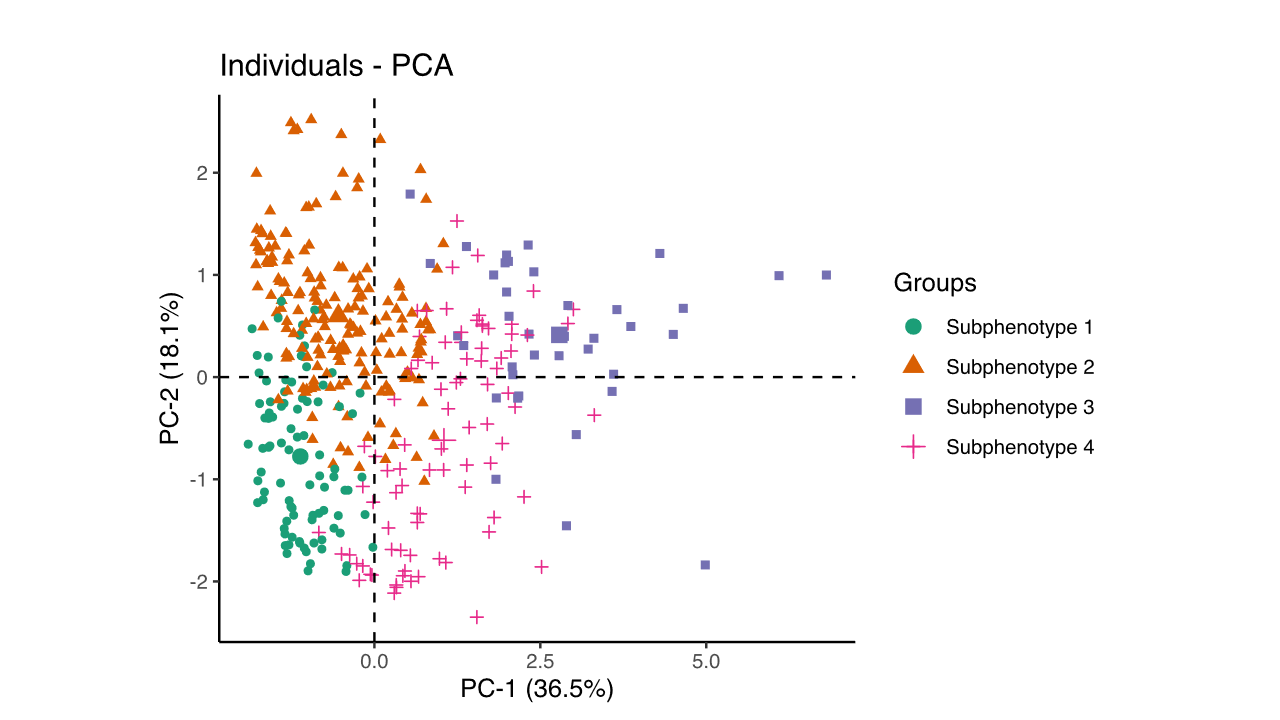

Supplement: Supplementary file 3 — Additional file 3. [file 13613_2024_1392_MOESM3_ESM.tif]

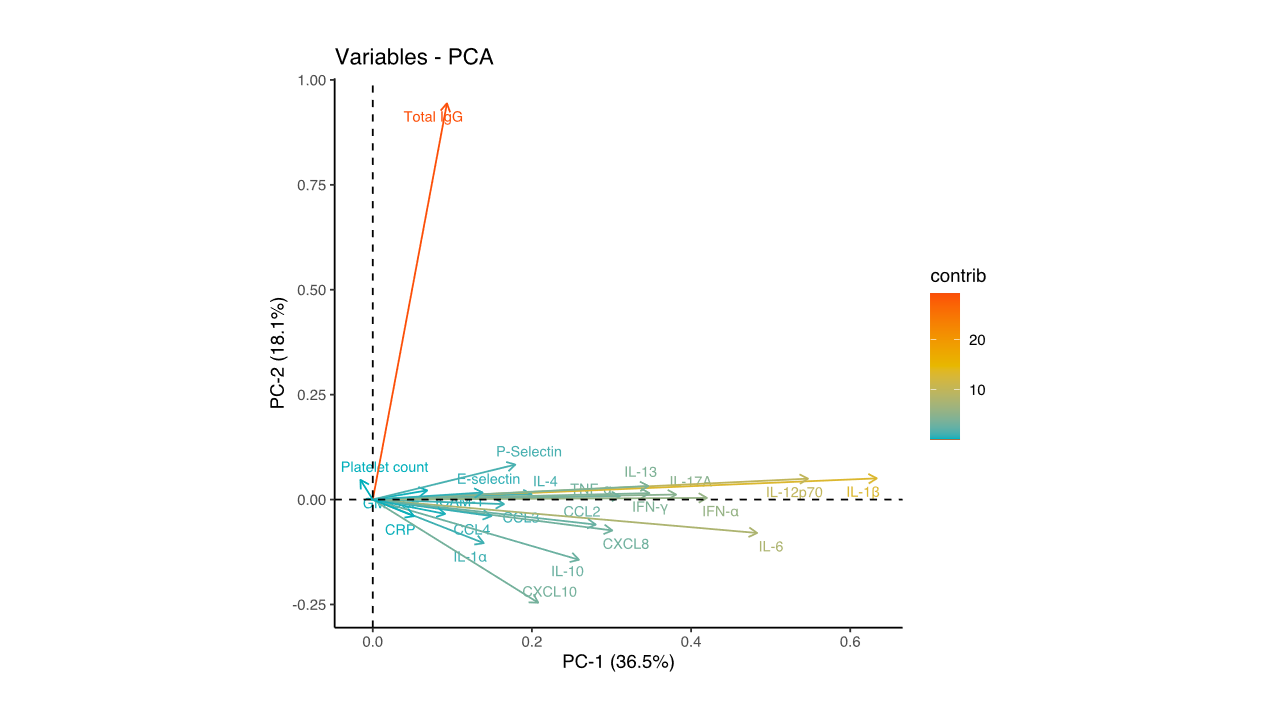

Supplement: Supplementary file 4 — Additional file 4. [file 13613_2024_1392_MOESM4_ESM.tif]
