# Supplementary material for: Immunological sub-phenotypes and response to convalescent plasma in COVID-19 induced ARDS: a secondary analysis of the CONFIDENT trial
Source: Ann Intensive Care. 2024 Oct 21;14:160. doi: 10.1186/s13613-024-01392-1 (PMC11493925; doi:10.1186/s13613-024-01392-1)
Supplement: Supplementary file 6 — Additional file 6. [file 13613_2024_1392_MOESM6_ESM.docx]

**Immunological sub-phenotypes and response to Convalescent Plasma in COVID-19 induced ARDS : a secondary analysis of the CONFIDENT trial.**

Supplemental Material

# **Authors**

Benoît Misset, MD (1), Anh Nguyet Diep, PhD (2), Axelle Bertrand, MSc (1), Michael Piagnerelli, MD, PhD (3), Eric Hoste, MD, PhD (4), Isabelle Michaux, MD, PhD (5), Elisabeth De Waele, MD, PhD (6), Alexander Dumoulin, MD (7), Philippe G Jorens, MD, PhD (8), Emmanuel van der Hauwaert, MD (9), Frédéric Vallot, MD (10), Walter Swinnen, MD (11), Nicolas De Schryver, MD (12), Nathalie de Mey, MD (13), Nathalie Layios, MD, PhD (1), Jean-Baptiste Mesland, MD (14), Sébastien Robinet, MD (1), Etienne Cavalier, EuSpLM, PhD (15) Anne-Françoise Donneau, PhD (2), Michel Moutschen, MD, PhD (16), Pierre-François Laterre, MD (17)

1: Department of Intensive Care Medicine, Liège University, CHU de Liège, Liege, Belgium

2: Biostatistic Unit, Public Health Department, Liège University, Liege, Belgium

3: Department of intensive care, CHU-Charleroi-Chimay Marie Curie Hospital, Université Libre de Bruxelles, Charleroi, Belgium

4: Department of Intensive Care Medicine, University Hospital, Gent, Belgium

5: Department of Intensive Care, Université Catholique de Louvain, CHU UCL Namur, Yvoir, Belgium

6: Department of Clinical Nutrition, Vrije Universiteit Brussel Brussels University Hospital, Jette, Belgium

7: Department of Intensive Care Medicine, Delta General Hospital, Roeselare, Belgium

8: Department of Intensive Care Medicine, Antwerp University Hospital, University of Antwerp, LEMP, Edegem, Belgium

9: Department of Intensive Care Medicine, Imelda General Hospital, Bonheiden, Belgium

10: Department of Intensive Care Medicine, Wallonie Picarde General Hospital, Tournai, Belgium

11: Department of Intensive Care Medicine, Sint Blasius General Hospital, Dendermonde, Belgium

12: Department of Intensive Care Medicine, Saint-Pierre General Hospital, Ottignies, Belgium

13: Department of Intensive Care Medicine, OLV General Hospital, Aalst, Belgium

14: Department of Intensive Care Medicine, Saint-Luc University Hospital, Brussels, Belgium

15: Department of Clinical chemistry, University of Liege, CIRM, CHU de Liège, Liege, Belgium

16: Department of Infectious diseases, CHU de Liège, Liege, Belgium

17: Department of Intensive Care Medicine, Mons-Hainaut Regional Hospital, Mons, Belgium

# **Correspondence**

Benoît Misset, MD,

Email address: [benoit.misset@chuliege.be](mailto:benoit.misset@chuliege.be)

## e-Table 2: Results of post-hoc pairwise comparisons regarding variables showing significant differences among the 4 subphenotypes

| **Time from ICU admission** | **Z-statistic** | **Adjusted p-value** |
| --- | --- | --- |
| Subphenotype 1 - Subphenotype 2 | -4.87 | <0.001 |
| Subphenotype 1 - Subphenotype 3 | -2.66 | 0.047 |
| Subphenotype 3 - Subphenotype 2 | -0.66 | 1 |
| Subphenotype 1 - Subphenotype 4 | -1.18 | 1 |
| Subphenotype 2 - Subphenotype 4 | 3.45 | 0.003 |
| Subphenotype 3 - Subphenotype 4 | 1.72 | 0.51 |
| **NP PCR test for SARS-Cov-2** | **Z-statistic** | **Adjusted p-value** |
| Subphenotype 1 - Subphenotype 2 | -1.23 | 1 |
| Subphenotype 1 - Subphenotype 3 | -0.34 | 1 |
| Subphenotype 3 - Subphenotype 2 | -0.60 | 1 |
| Subphenotype 1 - Subphenotype 4 | 1.90 | 0.34 |
| Subphenotype 2 - Subphenotype 4 | 3.51 | 0.003 |
| Subphenotype 3 - Subphenotype 4 | 1.93 | 0.32 |
| **APACHE II score (points)** | **Z-statistic** | **Adjusted p-value** |
| Subphenotype 1 - Subphenotype 2 | 1.03 | 1 |
| Subphenotype 1 - Subphenotype 3 | -0.92 | 1 |
| Subphenotype 3 - Subphenotype 2 | 1.74 | 0.49 |
| Subphenotype 1 - Subphenotype 4 | -2.77 | 0.03 |
| Subphenotype 2 - Subphenotype 4 | -4.20 | <0.001 |
| Subphenotype 3 - Subphenotype 4 | -1.24 | 1 |
| **SOFA total (points)** | **Z-statistic** | **Adjusted p-value** |
| Subphenotype 1 - Subphenotype 2 | 0.17 | 1 |
| Subphenotype 1 - Subphenotype 3 | -1.21 | 1 |
| Subphenotype 3 - Subphenotype 2 | 1.44 | 0.90 |
| Subphenotype 1 - Subphenotype 4 | -3.95 | <0.001 |
| Subphenotype 2 - Subphenotype 4 | -4.72 | <0.001 |
| Subphenotype 3 - Subphenotype 4 | -1.86 | 0.38 |
| ***Sofa_Respiratory*** | **Z-statistic** | **Adjusted p-value** |
| Subphenotype 1 - Subphenotype 2 | -0.89 | 1 |
| Subphenotype 1 - Subphenotype 3 | -3.70 | 0.001 |
| Subphenotype 3 - Subphenotype 2 | 3.36 | 0.005 |
| Subphenotype 1 - Subphenotype 4 | -4.10 | <0.001 |
| Subphenotype 2 - Subphenotype 4 | -3.84 | 0.001 |
| Subphenotype 3 - Subphenotype 4 | 0.50 | 1 |
| **Sofa_cardiovascular** | **Z-statistic** | **Adjusted p-value** |
| Subphenotype 1 - Subphenotype 2 | 0.18 | 1 |
| Subphenotype 1 - Subphenotype 3 | -1.22 | 1 |
| Subphenotype 3 - Subphenotype 2 | 1.45 | 0.88 |
| Subphenotype 1 - Subphenotype 4 | -3.12 | 0.01 |
| Subphenotype 2 - Subphenotype 4 | -3.77 | 0.001 |
| Subphenotype 3 - Subphenotype 4 | -1.21 | 1 |
| **FiO2** | **Z-statistic** | **Adjusted p-value** |
| Subphenotype 1 - Subphenotype 2 | -1.64 | 0.60 |
| Subphenotype 1 - Subphenotype 3 | -3.49 | 0.003 |
| Subphenotype 3 - Subphenotype 2 | 2.59 | 0.06 |
| Subphenotype 1 - Subphenotype 4 | -3.38 | 0.004 |
| Subphenotype 2 - Subphenotype 4 | -2.27 | 0.14 |
| Subphenotype 3 - Subphenotype 4 | 0.85 | 1 |
| **PaO2/FiO2** | **Z-statistic** | **Adjusted p-value** |
| Subphenotype 1 - Subphenotype 2 | 1.79 | 0.44 |
| Subphenotype 1 - Subphenotype 3 | 3.52 | 0.003 |
| Subphenotype 3 - Subphenotype 2 | 2.51 | 0.07 |
| Subphenotype 1 - Subphenotype 4 | 3.89 | 0.001 |
| Subphenotype 2 - Subphenotype 4 | 2.71 | 0.04 |
| Subphenotype 3 - Subphenotype 4 | -0.48 | 1 |
